# Natural‐History Mapping of Lysosomal Storage Disorders (LSDs): Gaucher Disease as a Model for Precision Care

**DOI:** 10.1002/jimd.70128

**Published:** 2026-01-12

**Authors:** Noor Ul Ain, Maya Vaishnaw, Pramod. K. Mistry

**Affiliations:** ^1^ Department of Internal Medicine Yale School of Medicine New Haven Connecticut USA; ^2^ Genetic Counseling Class of ′25 University of Michigan Ann Arbor Michigan USA; ^3^ Department of Internal Medicine and Pediatrics Yale School of Medicine New Haven Connecticut USA

**Keywords:** Gaucher disease, lysosomal storage disorders, multi‐state disease modelling, natural‐history studies

## Abstract

Natural‐history datasets have become pivotal for drug development and for shaping clinical‐practice guidelines in rare diseases, yet many lysosomal storage disorders would benefit from deep phenotyping and modern analytic methods. Our objective was to integrate the past decade of genomic, cellular, treatment‐outcome, and regulatory advances into a practical framework for capturing and interpreting natural‐history data, using Gaucher disease (GD) as a paradigm. We reviewed more than 300 peer‐reviewed articles (2005–2025), FDA guidance documents, and output from large real‐world registries. Particular attention was paid to long‐read GBA sequencing, biomarkers such as lyso‐Gb1, emerging newborn screening programs, and preliminary observational work that points toward multi‐state disease modeling. Key observations include: (i) Whole‐gene sequencing has expanded genotype–phenotype maps, revealing more than 70 recombinant GBA alleles that confound panel tests; (ii) registry trajectories suggest that formal multi‐state models could capture treatment‐modified courses and silent endpoints—monoclonal gammopathy, malignancy, Parkinson's disease, pulmonary arterial hypertension—better than current summary statistics; (iii) lyso‐Gb1 outperforms legacy biomarkers and now serves as a second‐tier newborn‐screening marker; (iv) Robust natural‐history evidence has already underpinned regulatory approvals across several lysosomal disorders—including olipudase alfa for ASMD, cerliponase alfa for CLN2, vestronidase alfa for MPS VII, and sebelipase alfa for infantile‐onset LAL‐D—demonstrating that well‐curated registries can serve as viable external controls for future LSD submissions. The convergence of deep phenotyping, genotype‐aware analytics, and systematic biomarker capture promises to transform natural‐history registries from descriptive archives into predictive engines. Gaucher disease offers a working template that, when extended across the LSD spectrum, can accelerate precision care and the development of next‐generation therapeutics.

**Trial Registration:** ClinicalTrials.gov identifier: NCT00358943, NCT03291223.

## 1 Introduction

Lysosomal storage diseases (LSDs) are rare, inherited disorders in which undegraded macromolecules accumulate within lysosomes and damage multiple organs [1]. Over the past two decades, our understanding of their biology and clinical course has expanded rapidly; yet, most reviews still rely on registry summaries or single‐center cohorts [2, 3, 4, 5, 6, 7].

Contemporary synthesis is timely. Updated FDA guidance on natural‐history data, long‐read sequencing that reveals complex alleles, deep‐phenotyping platforms, and multi‐state disease modeling are reshaping research priorities and regulatory expectations. Biomarker discovery has kept pace: serum glucosylsphingosine (GlcSph, also known as Lyso GL1 or LysoGb1) in Gaucher disease, for example, outperforms legacy markers and already serves as a second‐tier newborn‐screening assay.

Gaucher disease (GD) remains the archetype for these shifts. Its clinical trajectory has been reshaped by enzyme‐replacement and substrate‐reduction therapies (ERT and SRT), forcing clinicians to redefine what “natural history” means once treatment itself alters the disease course. The same registry‐based analytics that mapped those changes now informs both sides of translational medicine, guiding regulatory approvals for multiple lysosomal therapies, such as olipudase alfa for acid sphingomyelinase deficiency, cerliponase alfa for CLN2 Batten disease, sebelipase alfa for infantile‐onset lysosomal acid lipase deficiency, and vestronidase alfa for MPS VII. Because clinical trials and single‐center studies enroll only narrow phenotype slices, the broad, longitudinal perspective of registries has become indispensable. These real‐world datasets not only inform drug approvals but also shape practice guidelines on when to initiate therapy, how to assess response, and how best to screen for late‐emerging complications across the full spectrum of disease. This review integrates genomics, cellular‐immune pathology, and real‐world registry analytics into a unified framework for lysosomal diseases. We aim to give clinicians, researchers, and regulators a practical playbook that (i) flags emerging phenotypes early, (ii) deploys genotype‐ and biomarker‐guided monitoring with precision, and (iii) turns natural‐history insight into daily clinical decisions and next‐generation trials, and (iv) shows how registry data can be leveraged into basic and translational discoveries.

## 2 Defining the Natural History Study

The FDA defines a natural‐history study as an observational investigation that documents disease onset, progression, variability, and complications in untreated individuals. Such studies identify patient subgroups, establish meaningful clinical outcomes, and validate biomarkers, and can serve as external controls for trials [5].

Yet in practice, the “natural” course of Gaucher disease (GD) is rarely untouched. Three factors modify it profoundly:

- Historical interventions: Splenectomy, once routine for symptomatic splenomegaly, redirects glucosylceramide‐laden macrophages to the bone, liver, lung, and brain, thereby amplifying skeletal, hepatic, pulmonary, and neurologic complications [1, 2, 3].
- Timing of diagnosis and therapy: Early detection and prompt ERT or SRT reduce irreversible damage and improve prognosis [8].
- Depth of monitoring: Systematic surveillance for malignancies (e.g., multiple myeloma, hepatocellular carcinoma) and serial lyso‐Gb1 measurements enable risk stratification and timely therapeutic adjustment.
- A fourth layer comes from life‐stage goals: In GD, osteoporosis prevention is not just about adult bone density; achieving peak bone mass in childhood forestalls the fragility fractures seen later in life.

Incorporating these variables—past surgeries, diagnostic timing, biomarker surveillance, and life‐course objectives—transforms classic natural‐history studies into treatment‐modified natural‐history models. Such nuanced data gives a more accurate picture of real‐world disease, informing patient care and accelerating drug development.

## 3 Gaucher Disease: Genetics, Cellular Pathology, and Immunopathology

The most impactful natural history studies are those that tightly integrate genomic insights with cellular and molecular pathology of the disease. Frequently, significant gaps persist in understanding these fundamental biological mechanisms. However, astute clinical observations can catalyze mechanistic insights, as exemplified by the recognition of links between Gaucher disease (GD) and Parkinson's disease or GD‐associated gammopathies and multiple myeloma [4, 5].

**FIGURE 1:**
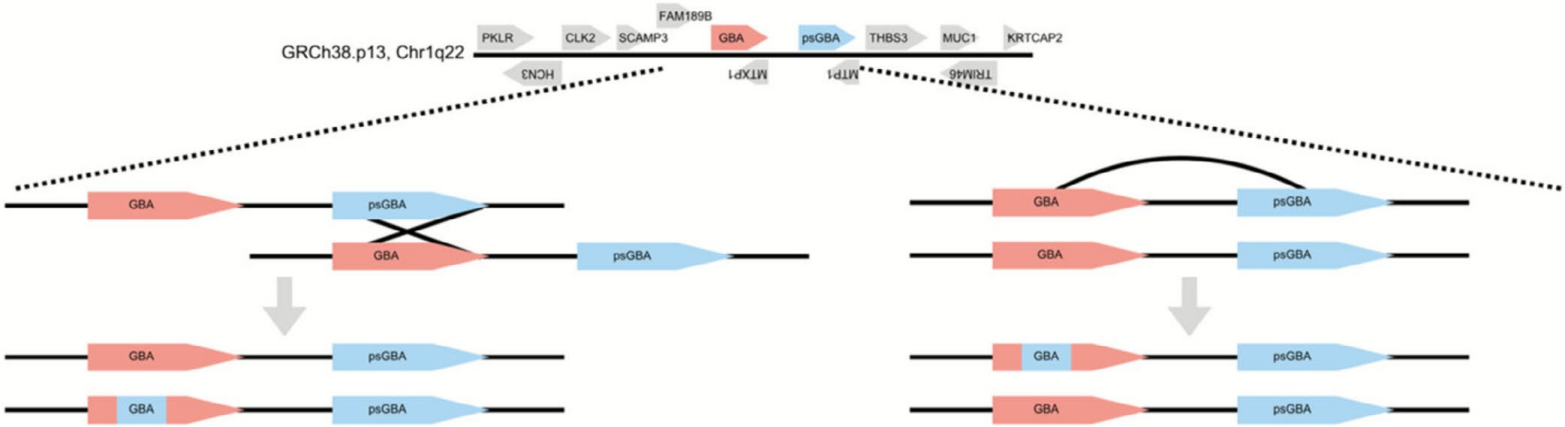
Complex GBA alleles arise from gene conversion events resulting from sequence homology with pseudogene psGBA. p.Asn409Ser mutation is not present in the pseudogene sequence but p.Leu483Pro is part of normal psGBA sequence. World‐wide p.Leu483Ser mutation is the most prevalent GBA mutation, underscoring the vulnerability of GBA locus to gene conversion events. (*Created in BioRender. Ain, N*. *2026* https://BioRender.com/9457cnb).

Gaucher disease arises from biallelic pathogenic variants in ***GBA***, situated within a gene‐dense segment of chromosome 1q21. The functional *GBA* gene lies immediately adjacent to a highly homologous pseudogene (*psGBA*), and this close, sequence‐rich pairing makes the locus particularly susceptible to gene‐conversion events that generate complex or recombinant alleles. These gene conversion events between *GBA* and *psGBA* frequently result in complex recombinant alleles that significantly influence clinical phenotypes and prognostic variability [7] (Figure 1).

The common *GBA* mutation * p.Asn409Ser *—still widely referred to by its legacy name *N370S* because most epidemiologic and historical papers pre‐date HGVS numbering—arose roughly 48 generations (≈1200 years) ago and is especially prevalent among Ashkenazi‐Jewish populations [8, 9]. This timing aligns closely with historical founder events and subsequent expansions of early medieval Ashkenazi Jewish communities. Importantly, this founder event suggests the intriguing possibility that additional genetic variants, co‐inherited alongside p.Asn409Ser, may significantly influence clinical variability and disease severity. Indeed, the same founder population is known to harbor variants associated with other genetic conditions such as Factor XI deficiency, Familial Mediterranean Fever (FMF), and Parkinson's disease, each potentially modifying the phenotypic expression and emerging clinical features of Gaucher disease within this specific population.

**FIGURE 2:**
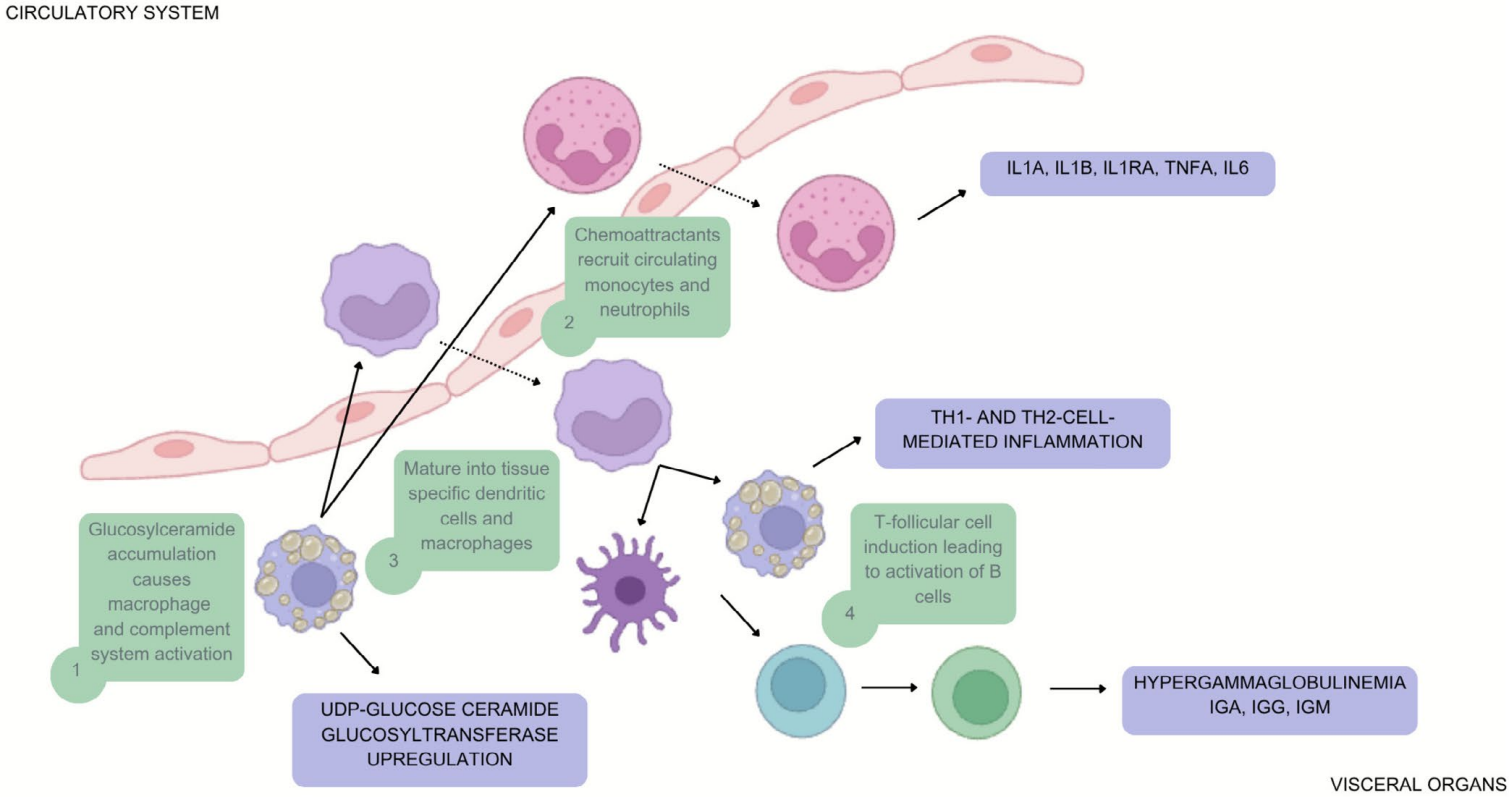
Overview of the immunological cascade in Gaucher Disease, with implications for systemic inflammatory response. Despite massive organomegaly seen in GD, the amount of GlcCer and GlcCer is barely 1% of organ weight. The accumulation of these immunoactive lipids, seen classically in lipid‐laden macrophages (Gaucher cells), elicits widespread immune activation and induction of UGCG, amplifying the metabolic defect [12, 14, 15, 16, 49] (*Created in BioRender. Ain, N*. (*2026*) https://BioRender.com/9457cnb).

At the cellular level, GD pathology extends far beyond macrophage involvement to affect almost every aspect of the innate and adaptive immune system. Biallelic GBA mutations and deficiency lysosomal glucocerebrosidase deficiency result in glucosylceramide (GlcCer) and glucosylsphingosine (GlcSph) accumulation within macrophages (Gaucher cells), with downstream pathology involving the dysfunction in diverse cell types, including neurons, osteoblasts, osteoclasts, and almost every other type of immune cell [10, 11, 12, 13, 14] (Figure 2). This expanded view of cellular pathology underlies the complex clinical spectrum, which ranges from protean skeletal disease, hepatic disease, lung disease, marrow disease, lymphatic system disorder, and neurodegeneration, as well as increased risk of hematologic and solid organ malignancies, such as hepatocellular carcinoma, renal cell carcinoma, and melanoma.

Hence, in Gaucher disease, there is profound immunopathological involvement mediated by the elevation of GlcCer and GlcSph, underpinned by chronic macrophage activation and systemic inflammation (Figure 2). This inflammatory milieu significantly impacts disease progression, symptoms (i.e., fatigue attributed to cytokine storm), and overall disease severity, ultimately driving comorbidities such as multiple myeloma. The context of genetic, cellular, and immunological underpinnings of Gaucher disease is thus essential for improved patient stratification, personalized therapeutic interventions, and optimization of clinical outcomes. Taken together, these insights define discrete disease nodes, that is, genetic, cellular, and immunological, that modulate the tempo of Gaucher progression and should guide both patient stratification and individualized intervention.

## 4 Historical Context and Controversies in Gaucher Disease Natural History and Registries

The first systematic Gaucher‐disease registry was launched by Robert Lee in Pittsburgh during the 1980s, and it revealed that even so‐called type 1 (non‐neuronopathic) patients could suffer severe complications: skeletal collapse, pulmonary arterial hypertension (PAH), liver failure, malignancies (myeloma, hepatocellular carcinoma), and Parkinson's disease [17]. Early cohorts, however, were skewed toward the sickest patients. Ernest Beutler noted that homozygosity for p.Asn409Ser (legacy N370S), recognized as a milder genotype, was strikingly underrepresented. He estimated that two‐thirds of such individuals might never reach medical attention and opined that selective enrollment could inflate both prevalence estimates and treatment use [18, 19].

Subsequent longitudinal studies, including ours, showed that p.Asn409Ser homozygotes are not benign; they accumulate progressive bone disease and carry a significantly higher lifetime risk of myeloma [6]. These data reset clinical expectations and underscore the need for lifelong surveillance. The International Collaborative Gaucher Group (ICGG) Registry (ClinicalTrials.gov, NCT00358943), launched in 1991 alongside the first ERT, Alglucerase and Imiglucerase, broadened the picture with thousands of global cases. Although its p.Asn409Ser cohort appeared milder on average, the registry confirmed age‐related complications similar to those seen in single‐center studies [20, 21]. More recently, other registries have been developed, such as the Gaucher Outcomes Survey (ClinicalTrials.gov ID NCT03291223) [10]. A major limitation of the GOS Registry is that it tends to lump all different therapies under generic ERT or SRT. This practice has hindered advances in clinical practice, precluding the prospect of conducting meaningful comparative effectiveness analysis of different therapies [22, 23]. Together, these registries illustrate both the power and pitfalls of natural‐history data: representation bias, evolving diagnostic criteria, and ethical debates over treatment thresholds. Ongoing refinements such as richer genotype–phenotype mapping, standardized recruitment, and deeper phenotyping continue to improve risk stratification and guide tailored therapy across the Gaucher spectrum.

## 5 Therapeutic Implications of Natural History Studies in Gaucher Disease

Natural‐history research has been the intellectual backbone of every significant therapeutic advance in Gaucher disease (GD). The earliest single‐center cohorts revealed the stark reality of unchecked visceral, skeletal, and hematological disease, providing clinicians with concrete endpoints, such as organ volumes, cytopenias, and sequelae of bone disease, against which to judge the first ERT trials. As the ICGG registry matured, its thousands of longitudinal patient‐years did more than chart progression; it revealed genotype‐specific trajectories, clarified the time course of complications such as avascular necrosis, osteopenia, osteonecrosis, and myeloma, and quantified dose–response relationships that shaped the “treat‐to‐target” paradigm [24]. Those same data supported regulatory filings that sustained improvements in spleen size, platelet count, and GlcSph (glucosylsphingosine, also referred to as lyso Gb1 or Lyso GL1) levels, which are clinically meaningful.

Crucially, natural‐history curves have also signaled where current therapies fall short. The persistence of neurological decline in neuronopathic GD, despite impeccable systemic control, spurred the search for brain‐penetrant small molecules and gene‐transfer vectors, now in early trials [18] Likewise, registry evidence of late‐onset malignancy, Parkinson's disease, and PAH reframed GD as a lifelong condition requiring age‐specific surveillance and periodic therapeutic recalibration. Therefore, by illuminating both what treatments can achieve and what they cannot, natural‐history studies have moved GD care from empirical dosing toward precision medicine, guiding when to start, how to monitor, and where to innovate next.

## 6 Early Diagnosis and Initiation of Therapy

Natural‐history studies leave little doubt that the longer Gaucher disease (GD) remains untreated, the greater the burden of irreversible damage and the burden of impaired quality of life. Although clinicians still base treatment starts on objective disease markers, genotype is now also integral to deciding how closely a patient is followed and when those markers trigger therapy, especially in the era of newborn screening.

Among patients with systemic disease, three genotype constellations deserve special attention. Children who carry one p.Asn409Ser (N370S) allele in trans with a null variant such as 84GG or IVS2 + 1 typically show rapid visceral enlargement, growth delay, and early skeletal disease manifestations. Experience shows that ERT or SRT initiation upon a sustained trend of GlcSph elevation, cytopenia, or splenomegaly can prevent avascular necrosis and osteoporosis and reverse established cytopenias and organomegaly [25, 26, 27]. A second high‐risk group comprises gene‐conversion alleles (RecNciI or p.Pro483Pro) often paired with p.Asn409Ser. These children present with marked hepatosplenomegaly, marrow infiltration, and recurrent bone crises in childhood; four‐ to six‐monthly surveillance and early treatment avert irreversible marrow and skeletal disease [28, 29]. Finally, patients who are homozygous for p.Leu483Pro (L444P) or who carry L444P in combination with a complex recombinant allele almost invariably evolve toward neuronopathic disease, although the time of onset varies widely. They still benefit dramatically from prompt systemic therapy with marked improvement of growth parameters, blood counts, and organomegaly. Additionally, these patients require neuro‐ophthalmic and cognitive monitoring and early enrolment in trials of brain‐penetrant agents [19].

The neuronopathic forms themselves (types 2 and 3) illustrate how timing can be everything. In infancy, the blood–brain barrier is relatively permeable and may offer a narrow window to ameliorate neurodegeneration, but it will not halt eventual neurodegeneration; yet, it often prolongs life and restores visceral/hematological health, buying precious time until CNS‐directed treatments mature [14, 30]. Because GBA gene‐conversion events appear to be common in the human genome, hence seen throughout the world, these severe genotypes may, in fact, outnumber the classic p.Asn409Ser‐homozygous GD1 population, reinforcing the public‐health value of newborn screening for early stratification.

Taken together, these observations suggest a genotype‐informed but evidence‐based strategy: do not treat on genotype alone, yet let genotype determine how intensively a child is monitored and how low the threshold for intervention should be. When subtle biochemical or clinical signs appear in a high‐risk genotype, acting early can convert GD management from belated damage control to genuine disease modification—ensuring normal growth, the attainment of peak bone mass, and a life unburdened by preventable complications.

## 7 Range of GBA Genotypes and Natural History in Gaucher Disease

**FIGURE 3:**
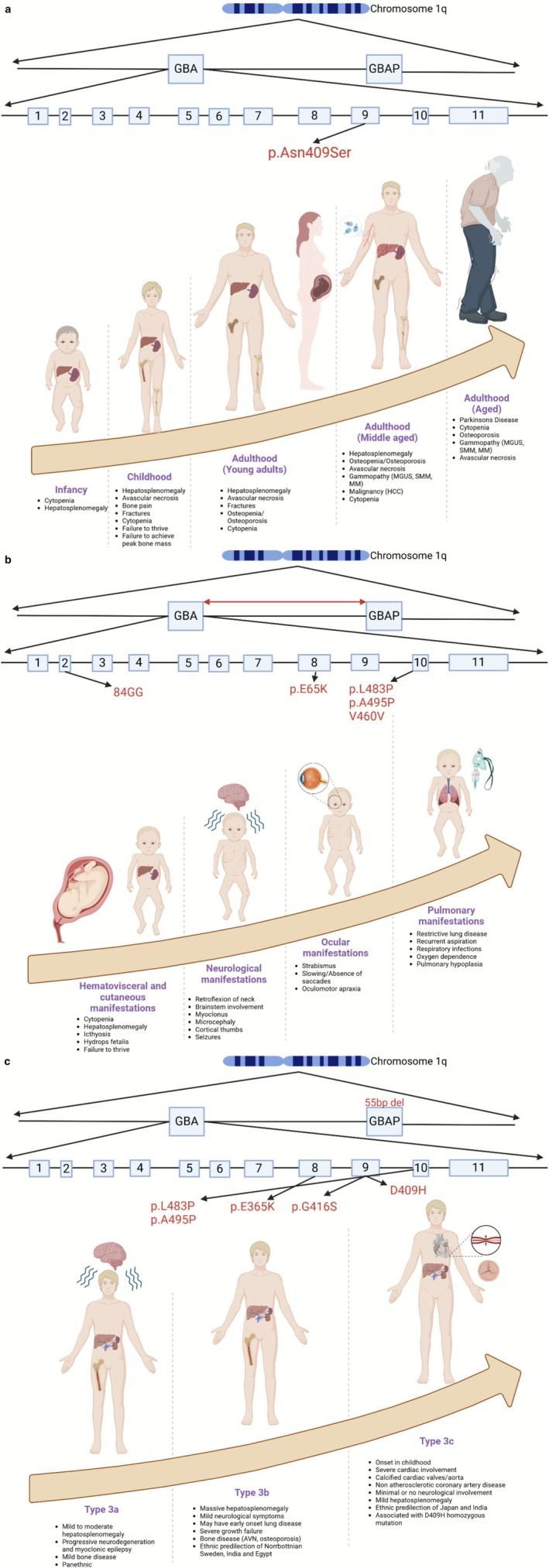
(a) Natural history of Gaucher disease: Systemic manifestations across the lifespan. (Cre*ated in BioRender. Ain, N*. (2026) https://BioRender.com/9457cnb). The p.Asn409Ser (N370S) variant in GBA, the most common pathogenic variant in Gaucher disease type 1, is shown within the gene's exonic structure. The lower panel depicts the age‐associated progression of systemic involvement in GD1, from infancy through older adulthood. Infants and children commonly present with hepatosplenomegaly, cytopenia, bone crises, and growth failure. In adulthood, persistent or worsening bone disease, gammopathy, and hepatobiliary complications, including hepatocellular carcinoma, emerge. Aged individuals are at increased risk of Parkinson's disease, osteoporosis, and progression to multiple myeloma. This trajectory emphasizes the chronic and evolving burden of Gaucher disease despite being considered “non‐neuronopathic.” (b) Phenotypic features of neuronopathic Gaucher disease: Perinatal to early childhood presentation. This figure illustrates the spectrum of Gaucher disease type 2, associated with severe mutations such as 84GG, p.E65K, p.L483P, p.A495P, and V460V. Manifestations begin prenatally or in early infancy with hematologic (cytopenia), visceral (hepatosplenomegaly), and cutaneous (ichthyosis) signs. Rapid neurologic deterioration follows, including brainstem dysfunction, cortical thumb posturing, and seizures. Ocular motor apraxia and pulmonary complications such as restrictive lung disease and pulmonary hypoplasia are also prevalent. The high lethality and multisystem involvement underscore the urgency for early diagnosis and experimental therapies. (c) Subclassification of neuronopathic Gaucher disease type 3: Genotype–phenotype correlations. Three phenotypic variants of type 3 Gaucher disease are shown, each linked to characteristic GBA mutations. Type 3a (e.g., p.L483P/p.A495P) is associated with progressive neurodegeneration and epilepsy. Type 3b (e.g., p.E365K/p.G416S) features massive visceral involvement, skeletal disease, and early pulmonary symptoms. Type 3c, strongly associated with the D409H mutation in homozygosity and a 55‐bp GBAP deletion, presents with severe cardiovascular manifestations including calcified cardiac valves and non‐atherosclerotic coronary disease. This figure illustrates the phenotypic heterogeneity of type 3 GD and the utility of genotype‐based subclassification for clinical management and monitoring.

The clinical course of GD is powerfully shaped, but not fully dictated, by the underlying GBA mutation. Figure 3 distills four decades of genotype–phenotype research into a lifespan narrative. Panel A follows the trajectory of a patient with at least one allele of p.Asn409Ser (N370S) that defines most type 1 GD: hepatosplenomegaly and cytopenias dominate childhood, skeletal disease and failure to reach peak bone mass appear in adolescence. Patients homozygous for this mutation demonstrate a continuing risk of bone complications despite relatively mild visceral and hematological disease. With age, these patients are at risk of Parkinson's disease, MGUS, and cancers [20, 21]. Most literature on type 1 GD is centered on this founder mutation, but other type 1 GD mutations are emerging around the world, which are likely to be more severe and lead to a more severe GD1 trajectory. Panel B captures the relentless arc of severe gene‐conversion alleles such as p.Leu483Pro or RecNciI. Here, visceral enlargement and restrictive lung disease surface in the first weeks of life, only to be eclipsed by fulminant neurodegeneration that claims most children with type 2 disease before their second birthday. Panel C depicts type 3 GD and shows that even within “slowly progressive” neuronopathic GD there is striking heterogeneity: the classic p.Leu483Pro pattern presents with variable childhood or adult onset of neurological disease, and massive systemic burden due to hepatosplenomegaly and cytopenia. Of note, the most consistent genotype/phenotype correlation is type 3c GD due to homozygosity of D409H (p.Asp448His) mutation with a distinctive course of relatively mild neurological symptoms, hydrocephalus, mild to moderate organomegaly but striking cardiac phenotype involving progressive calcification of valves and aorta associated with non‐atheromatous coronary artery disease that can lead to sudden death despite otherwise adequate systemic ERT. The Figure 3 panels make three clinical lessons unmistakable. First, genotype sets the tempo but not the entire script: N370S homozygotes can still develop myeloma or avascular necrosis, while some L444P homozygous patients progress neurologically very slowly [25, 26]. Second, the organ systems at greatest risk shift with age; the visceral‐hematological burden of childhood gives way to bone, malignancy, neurological (PD in adults), or cardiac (in D409H in type 3c) complications in adults. Third, surveillance should include genotype‐specific and age‐specific perspectives. A child with D409H homozygosity needs annual echocardiography alongside neurological review, whereas an N370S/null teenager warrants early bone‐density tracking, visceral/hematological/growth assessments with a focus to prevent splenectomy.

## 8 Patient Stratification and Personalized Care

Natural history modelling enables the management of Gaucher disease (GD) to transition from a “one size fits all” approach to genotype‐ and trajectory‐based care. Longitudinal data show that a small fraction of p.Asn409Ser (N370S) homozygotes can remain pauci‐symptomatic for decades; for these patients, vigilant monitoring, rather than immediate therapy, can be appropriate [27]. Crucially, deep phenotyping is still required because conventional bedside markers such as spleen size do not capture hidden skeletal burden or the age‐related risks of malignancy and Parkinson's disease.

At the other end of the spectrum, genotypes that consistently predict neuronopathic involvement, for example, p.Leu483Pro (L444P) homozygosity or complex L444P recombinants, demand early ERT plus regular neuro‐ophthalmic review. Even when visceral disease responds, treaters must anticipate extrasplenic complications such as massive intra‐abdominal lymphadenopathy, chest/spinal deformities, or progressive lung involvement and adjust therapy (e.g., add SRT or combination regimens) as soon as they surface [6]. Genotype therefore guides the cadence and focuses surveillance, but individual trajectory refines the therapeutic goal. Baseline marrow MRI, liver‐ and spleen‐volume measurements, DXA scans, blood counts, and GlcSph help map that trajectory; repeated at defined intervals, they tell the clinician when to escalate, switch, or taper treatment. Finally, certain genotypes warrant organ‐specific precautions. Children who are p.Asp448His (D409H) homozygous, for example, require routine echocardiography and coronary imaging because calcified valves and non‐atheromatous coronary disease remain the leading causes of death even in childhood despite adequate systemic therapy. Likewise, the subset of p.Leu483Pro patients prone to severe spinal or chest‐wall deformity benefit from early orthopedic referral and bracing to slow progression [28]. It will be interesting to watch the natural history of this devastating complication in the era of brain‐penetrant SRT. Hence, a multi‐pronged approach—melding genotype data, deep phenotyping, and natural‐history analytics—enables truly personalized Gaucher care: low‐risk patients can be spared overtreatment, while high‐risk genotypes receive targeted surveillance and timely therapeutic adjustments.

## 9 Management of Age‐Related Manifestations and Comorbidities

Long‐term natural history studies make it clear that Gaucher disease does not “fade out” once visceral and hematological targets are brought under control with ERT. Instead, the disease resurfaces in later decades with a new cast of complications—monoclonal gammopathy and myeloma, other hematological malignancies, solid‐organ cancers, new avascular‐necrosis or osteoarthritis complications of old AVN, Parkinson's disease, and PAH. These late events follow a predictable age gradient, yet they often remain invisible until they produce irreversible harm. Modern management, therefore, demands a lifespan framework that layers age‐specific surveillance and pre‐emptive intervention on top of the traditional spleen‐liver‐bone targets established in childhood and young adults. The sections that follow translate natural‐history curves into concrete monitoring schedules and therapeutic pivot points for each of these age‐linked threats.

## 10 Bone Health Across Lifespan

Longitudinal registries demonstrate that the attainment of low peak bone mass persists beyond childhood growth; rather, it becomes a lifelong concern, culminating in fragility fractures if left unrecognized. Achieving peak bone mass during adolescence is therefore a key therapeutic goal, as low gains in those years can directly translate into later fractures. The link is both biological and epidemiological: GlcSph—now a validated GD biomarker—is toxic to osteoblasts, providing a mechanistic bridge between lysosomal dysfunction and impaired bone formation [15, 29, 30].

Routine dual‐energy X‐ray absorptiometry, targeted MRI for marrow infiltration or early avascular necrosis, and serial GlcSph measurements guide decisions about escalating ERT or adding adjunctive therapy. Notably, homozygosity for p.Asn409Ser presents a paradox: minimal visceral disease but a disproportionate risk of osteopenia and AVN in mid‐life and beyond. Patients with non‐p.Asn409Ser genotypes face an even higher prevalence of osteonecrosis and low bone density, accompanied by significant visceral and hematologic disease, underscoring the need for genotype‐aware surveillance and early orthopedic input as needed [20]. Importantly, focal lytic lesions should not be assumed to represent Gaucher bone involvement; rapidly enlarging defects, particularly in patients with emerging monoclonal gammopathy, may herald myeloma or another malignancy mislabeled as “Gaucheroma” and therefore warrant full oncologic evaluation.

## 11 Myeloma and Other Cancers

Chronic lipid‐antigen presentation in Gaucher disease keeps macrophages and antigen‐presenting cells in a state of low‐grade activation; over time, this drives T‐cell exhaustion, tilting immune surveillance toward tolerance rather than tumor clearance [13]. The clinical footprint of that immune dysregulation extends well beyond monoclonal gammopathy and myeloma. Population‐matched registry analyses now show a two‐ to four‐fold excess of hepatocellular carcinoma, renal‐cell carcinoma, and cutaneous melanoma in adults with type 1 GD, cancers that share an immuno‐oncologic profile in which exhausted or senescent T cells play a permissive role [31]. For the practicing clinician, any new focal lesion—hepatic, renal, cutaneous, or skeletal—should therefore be approached with heightened suspicion.

## 12 Parkinson's Disease

Natural‐history analyses beginning with a large Yale/NYU cohort first quantified the burden of Parkinson's disease in type 1 Gaucher disease (GD). In that study, 11 out of 444 well‐phenotyped patients with type 1 GD developed clinically overt Parkinsonism, a prevalence ≈20‐fold higher than in matched controls and with a mean age at onset a decade earlier than idiopathic PD. Later registry work confirmed similar figures: by age 60, one in nine GD1 patients meets PD criteria, and by age 80, nearly one in five will have PD, DLB, or a related movement disorder.

Mechanistic data explain this risk elevation. GlcSph, the signature bioactive lipid of GD, directly seeds and stabilizes toxic α‐synuclein oligomers, accelerating Lewy pathology [32]. Genetic modifiers provide a second layer: a multicentre analysis demonstrated that a common‐variant polygenic PD risk score stratifies parkinsonism risk even within Gaucher cohorts, with higher scores predicting earlier phenotype conversion [33].

Taken together, these data are shifting clinical practice. From age 40 onward, GD1 patients should undergo systematic screening for prodromal PD features such as hyposmia and REM‐sleep behavior disorder. Eventually, those at elevated polygenic or biomarker risk will be prioritized for brain‐penetrant SRT or α‐syn‐modifying trials. Multidisciplinary collaboration with movement‐disorder specialists ensures that motor symptoms are detected early. Gaucher disease is thus no longer viewed as a purely systemic lysosomal disorder; it is recognized as a lifelong condition whose neurologic complications are predictable, quantifiable, and in the future will be potentially modifiable.

## 13 Splenectomy—An Intervention with Long‐Term Risks

Before ERT became available, splenectomy was often the only way to control massive splenomegaly and life‐threatening cytopenias in Gaucher disease. Longitudinal registries have since shown that removing the spleen does far more than eliminate a source of hypersplenism. Asplenic patients carry a markedly higher incidence of avascular necrosis, cancers, advanced liver fibrosis, and pulmonary arterial hypertension (PAH) [8, 34, 35, 36, 37, 38]. There has been debate about whether splenectomy was a marker of disease severity, that AVN would occur whether or not splenectomy was performed. There is support for the latter concept from the UK cohort.

The PAH story illustrates how natural‐history data can correct misperceptions. Early case reports blamed ERT for precipitating PAH; some centers even recommended stopping therapy when PAH emerged [39]. Subsequent single‐center registry analyses showed the opposite: splenectomy is the dominant risk factor, whereas continued ERT or substrate‐reduction therapy stabilizes—and in some cases reverses—hemodynamic decline. Patients who discontinued ERT deteriorated rapidly (Mistry PK, unpublished observations), underscoring the danger of attributing post‐splenectomy complications to the very treatment that mitigates them [40].

These findings have reshaped modern practice. Splenectomy is now reserved for exceptional circumstances, and lifelong, organ‐specific surveillance is mandatory for already asplenic patients: periodic liver elastography and imaging for fibrosis or HCC, bone‐density and marrow assessments for early AVN, and routine echocardiography or right‐heart catheterization when indicated to detect evolving PAH. Recognizing splenectomy as a modifiable risk factor—and maintaining ERT/SRT in those who have already lost their spleen—has become a cornerstone of contemporary Gaucher management. A deeper appreciation of the long‐term consequences of asplenia has driven a dramatic reduction in splenectomies among the current generation of Gaucher patients; registry analyses confirm a striking drop in splenectomy rates in the post‐ERT era [41].

## 14 What Large Registries have Changed—And Why It Matters

More than two decades of longitudinal data from global lysosomal‐disease registries (≈18 500 patients and > 700 000 person‐years across Gaucher, Fabry, Pompe, and MPS I) have shifted evidence standards from anecdote to population‐level analytics. (Table 1) The International Collaborative Gaucher Group (ICGG) Registry alone contributes over 260 000 patient‐years and has:

- Mapped genotype–phenotype spectra far beyond single‐center ancestry biases.
- Clarified modifiable risk factors for bone crises, avascular necrosis, and malignancy;
- Demonstrated that splenectomy—rather than ERT—is a major determinant of severe bone complications, adding to other studies of the association of splenectomy with PAH and progressive liver disease.
- Provided real‐world dose–response data that underpin current “treat‐to‐target” algorithms [42].

**TABLE 1:** Total number of patients and person‐years in rare disease registries.

Registry	Year registry was established	Total countries*	Total registry sites	Total patients	Total person‐years from birth to last follow‐up	N	Total person‐years from diagnosis to last follow‐up	N	Total person‐years from treatment initiation to last follow‐up	*N*
ICGG Gaucher registry	1991	64	278	6872	266 543	6844	112 115	6481	67 470	5595
Fabry registry	2001	47	243	7930	344 445	7897	78 220	7267	38 523	5017
MPS I registry	2003	41	144	1325	18 598	1323	13 497	1297	10 086	1176
Pompe registry	2004	47	240	2467	87 251	2463	21 761	2405	13 510	2210
**Total**			**805**	**18 594**	**716 837**	**18 527**	**235 593**	**17 450**	**129 589**	**13 798**

Because registry cohorts capture the full heterogeneity of Gaucher disease, including extremes rarely seen in clinical trials, their outputs now guide everyday decisions. For example, when to start or escalate ERT/SRT, how to surveil for Parkinsonism, myeloma, hepatocellular carcinoma, and when to add adjunctive bone or cardiopulmonary monitoring. They have also supplied the external‐control datasets that regulators used to approve therapies for ASMD, infantile‐onset Pompe, and other ultra‐rare disorders, upgrading well‐curated real‐world evidence from “supportive” to “pivotal” in drug development. (Table 2) Together, the registries have become both the lens through which we view natural history and the backbone of precision care. Their value lies less in headline enrolment numbers than in the continuous, structured follow‐up that converts heterogeneous clinical reality into actionable standards. Herein, these standards are embedded throughout this review's recommendations on genotype‐informed surveillance, age‐specific monitoring, and therapy optimization.

**TABLE 2:** Comparison of Natural History Data in LSDs.

Disease	Typical onset	Key biomarkers	Natural history studies	Therapeutic advances
Gaucher	Childhood to Adulthood	Lyso‐GL1, chitotriosidase	ICGG, French registry, UK Gaucherite	ERT, SRT, gene therapy trials
Fabry	Adolescence to adulthood	Gb3, lyso‐Gb3	Fabry Registry	ERT, chaperone therapy
Pompe	Infantile to adult	GAA enzyme levels	Pompe Registry	ERT, gene therapy trials
Niemann‐Pick Type C	Infantile to adult	Oxysterols, lysosphingomyelin	Emerging studies	Miglustat, experimental therapies
MPS	Infancy to early childhood	GAG levels (urinary), Enzyme assays	MPS registries (Hunter Outcome Survey, MPS I registry)	ERT, HSCT, experimental gene therapy

## 15 Leveraging Natural History Data to Personalize Patient Education in Gaucher Disease Genetic Counseling

Genetic counseling education in Gaucher disease has evolved from a simple explanation of autosomal‐recessive inheritance into a nuanced, lifespan‐oriented dialog that draws heavily on natural‐history data. Registries now make clear that prognosis hinges on both genotype and age: homozygosity for p.Asn409Ser (N370S) generally spares the central nervous system yet carries a later‐life burden of osteonecrosis, malignancy, and Parkinson's disease; p.Leu483Pro (L444P) homozygosity almost invariably presages neuronopathic Gaucher, but with widely variable age of onset and severity. Homozygosity for p.Asp448His (D409H) mutation leads to a distinctive phenotype, dominated by aortic and valvular calcification and proximal non‐atheromatous coronary artery disease. While these correlations may be valuable to patients, they must be tempered by the variability revealed in sibling pairs and monozygotic twins, so counselors emphasize both the predictive power and the limitations of genotype‐based forecasts [43] (Table 3).

**TABLE 3:** What natural‐history data add to modern genetic counseling.

Counseling domain	Registry‐driven insight	Practical takeaway
Prognosis by genotype	N370S homozygosity usually spares the CNS but still carries age‐linked risks (osteonecrosis, malignancy, PD); L444P homozygosity predicts neuronopathic disease; D409H homozygosity leads to type 3c with cardiac phenotype.	Offer genotype‐specific surveillance schedules and clarify the limits of prediction (twin‐twin discordance, modifier genes).
Recombinant alleles	Gene‐conversion events (RecNci I, RecTL) can shift a seemingly “mild” genotype into a severe phenotype.	Ensure full‐length GBA sequencing/long‐template PCR and warn families of test‐interpretation pitfalls.
Carrier issues	Heterozygous GBA carriers have a 3–5‐fold PD risk; polygenic PD scores and lipid biomarkers refine this further.	Discuss PD surveillance, lifestyle factors, and research trials; reassure that most carriers remain disease‐free.
Psychosocial & cultural context	Studies highlight uncertainty, stigma, and decision‐making stress in tight‐knit or high‐consanguinity communities.	Provide culturally sensitive resources, peer networks, and mental‐health referrals.

The counseling session also addresses the technical caveats of testing. Recombinant alleles generated by gene conversion between GBA and its pseudogene can transform an apparently “mild” genotype into a severe clinical course, yet they are easy to miss on standard multigene panels [34]. Families therefore need to understand why full‐length GBA amplification in an experienced laboratory is essential and how a second opinion can avert diagnostic error.

Because every child with Gaucher disease has two carrier parents, the counselor's remit extends naturally from the affected proband to the entire family constellation. Occasionally, in Ashkenazi Jewish families, one of the parents is affected due to the high gene frequency in this population. Each obligate heterozygote shares the 3–5‐fold excess risk of Parkinson's disease that recent natural‐history studies have confirmed in GBA carriers. Common‐variant polygenic scores can sharpen that estimate still further, identifying relatives who might benefit most from prodromal screening once they reach their forties, from lifestyle measures that support dopaminergic resilience, and from enrolment in prevention trials designed to lower central glucosylsphingosine [35].

Genetics education, however, is only one part of the conversation and must be informed by the psychosocial lens. In communities where consanguinity is customary or where genetic diagnoses carry social stigma—among Orthodox Ashkenazi Jews, for example, or in parts of the Middle East and North Africa—families may worry about diminished marriage prospects, community standing, or anxiety spreading through extended kin networks. Comprehensive counseling, therefore, pairs medical guidance with culturally attuned support, offering facilitated disclosure strategies, referrals to peer groups and mental‐health services, and clear explanations of reproductive options ranging from expanded carrier screening to prenatal or pre‐implantation testing. In this family‐centered model, the counselor is not only an educator but also an individualized support, helping each relative translate shared genetic risk into informed, individually appropriate choices across the lifespan.

By weaving natural‐history evidence into conversations about prognosis, test limitations, carrier risk, and psychosocial context, genetic counselors can help turn abstract molecular data into a personalized health roadmap—one that not only anticipates challenges but actively empowers families. For carriers of pathogenic GBA variants, for example, this means translating elevated Parkinson's disease risk into actionable steps: adopting regular aerobic and resistance exercise, optimizing cardiovascular health, ensuring restorative sleep, and joining research registries that offer early‐access monitoring. It also means building a lifelong surveillance plan that extends beyond the annual physical: structured non‐motor screening for hyposmia, REM sleep behavior disorder, constipation, and anxiety, starting in mid‐adulthood, coupled with periodic motor examinations or digital gait assessments to detect subtle bradykinesia. In this way, counseling education shifts from passive communication of inheritance and risk to proactive support, working with each family to develop a concrete strategy for living well with a GBA carrier state while science advances toward definitive prevention [36].

## 16 Future Directions: From Natural‐History Curves to Dynamic Precision Medicine

The next leap in Gaucher‐disease research will come from linking long‐running natural‐history registries with multi‐state models that view this chronic illness as a sequence of clinically recognizable milestones rather than a single, linear trajectory. By assigning each patient to a state—minimal disease, early bone involvement, advanced skeletal fragility, malignancy, Parkinsonism, and so on—and calculating the probability and timing of transitions, these models turn decades of observational data into individual risk forecasts. In practice, that means knowing, for example, when an asymptomatic p.Asn409Ser homozygote is likely to develop osteonecrosis, or how soon a child with a gene‐conversion allele might progress from subtle horizontal saccades to neurological disability or myoclonic epilepsy.

Multi‐state outputs become more powerful when they combine real‐world inputs in real time: serial serum GlcSph levels, MRI marrow scores, wearable‐derived gait metrics, even natural‐language symptom snippets mined from clinic notes. Machine‐learning overlays can refine transition probabilities in real time, while genomic and polygenic‐risk layers provide a personalized baseline. It is envisioned that the result is a living decision aid that alerts clinicians to initiate or escalate therapy before a complication becomes irreversible, designs adaptive trial end‐points around the most meaningful state changes, and supplies regulators with granular external controls.

Although the most mature datasets originate from GD, the same framework is immediately portable to Fabry, Pompe, and MPS I, where registries already catalog the stepwise emergence of renal, cardiac, respiratory, or neurological disease. With each iteration, multi‐state modeling pushes lysosomal‐storage disorders closer to a truly precision‐medicine paradigm—one in which treatment is not merely initiated but continuously tailored to each patient's evolving risk landscape.

## 17 Registries as Engines of Drug Approval

The precedent is now clear: carefully curated natural‐history datasets can serve as the primary evidence base for marketing authorization when randomized trials are unfeasible. Table 4 lists six lysosomal disorders whose therapies reached the FDA and/or EMA primarily on the strength of external natural‐history cohorts. In each case, those data either supplied a matched comparator for single‐arm studies (as with cerliponase alfa in CLN2 and olipudase alfa in ASMD) or established baseline trajectories against which open‐label results were judged clinically meaningful—for example, the 12‐month survival advantage that secured sebelipase alfa approval in infantile LAL‐D.

**TABLE 4:** Therapies approved with pivotal support from natural‐history data.

Disorder (gene)	Therapy (year first approved)*	Role of natural‐history evidence
ASMD/SMPD1	Olipudase alfa (2022)	Baseline DL_CO, spleen volume and platelet trajectory from a multi‐center cohort enabled single‐arm efficacy trials [44].
CLN2/TPP1	Cerliponase alfa (2018)	Treated children were compared with a matched historical cohort on the CLN2 motor‐language score, demonstrating a clear divergence in slope [38].
MPS VII/GUSB	Vestronidase alfa (2017)	Open‐label study outcomes in 6‐min‐walk and pulmonary function were judged against registry‐derived floor data [45].
LAL‐D/LIPA	Sebelipase alfa (2015)	Twelve‐month survival was compared with an historical infant cohort (79% vs. 0%), obviating the need for a control arm [46].
Pompe, infantile‐onset/GAA	Alglucosidase alfa (2006)	Dutch–US natural‐history dataset served as the external comparator for ventilator‐free survival. [47]
MPS II/IDS	Idursulfase (2006 EU; 2007 US)	Registry data justified a ≤ 10% change in 6‐min‐walk distance and FVC as clinically meaningful end‐points [48].

* Year refers to first FDA or EMA marketing authorization.

Regulators have accepted such evidence for four overlapping reasons. First, placebo control is ethically untenable in ultra‐rare, rapidly progressive diseases such as infantile Pompe or CLN2. Second, the tiny populations involved make conventional randomization statistically underpowered and logistically impractical; matched historical cohorts offer the only realistic alternative. Third, the registries themselves—prospectively collected, protocol‐driven, and rich in standardized endpoints—provide data quality that can potentially inspire confidence: longitudinal global registry did so for ASMD, and the DEM‐CHILD network did the same for CLN2 [37, 38]. Finally, the outcomes tracked in these datasets—organ volumes, pulmonary function, ventilator‐free survival, validated functional scales—are objective, reproducible, and easily compared across cohorts.

The implications for future lysosomal drug development are straightforward. Prospective, well‐annotated registries remain indispensable, especially for genotype‐defined or survival‐indexed phenotypes in which classic trials will never be large. Modern analytical tools—such as propensity weighting, Bayesian borrowing, and synthetic controls—can further enhance the statistical efficiency of these datasets while meeting evolving regulatory requirements. As biochemical markers, for example, serum GlcSph levels in Gaucher disease or oxysterols in Niemann–Pick C, are integrated into natural‐history curves, sponsors will be able to directly link surrogate reductions to historically documented clinical milestones, thereby strengthening claims of disease modification. Together, these precedents show that high‐quality natural‐history evidence, collected with regulatory use in mind, can shorten development timelines and bring lifesaving therapies to patients who would otherwise remain untreated.

## 18 Future Directions and Conclusions

The next breakthroughs in lysosomal‐disease care will come from a community‐wide, collective effort: treat natural‐history data as a living dynamic infrastructure, not an archival record. Global registries linked in real time to electronic health records, wearables, and biobanks can feed machine‐learning models that forecast an individual's risk of bone crisis or neurodegeneration. Adaptive trial platforms could then utilize those forecasts to enroll the right patient at the right moment, testing brain‐penetrant small molecules, gene therapies, or immune modulators against endpoints that have been validated by decades of observation. Each success will, in turn, flow back into the registries, tightening the feedback loop between discovery and care.

Achieving that vision demands more than technology; it requires sustained international collaboration, shared data standards, and equitable access so that insights generated are applicable across countries. If the lysosomal‐disease community rises to that challenge, natural‐history studies will fulfil their highest purpose: not simply charting the course of illness, but actively bending it—patient by patient—toward a future where diagnosis is swift, therapy is precisely tailored, daily quality of life is meaningfully restored, and life expectancy nears the population norm. That goal is within reach; the infrastructure is already being built. What remains is the collective will to finish the job and deliver its benefits to every family living with these once‐intractable disorders.

## Funding

The authors have nothing to report.

## Conflicts of Interest

The authors declare no conflicts of interest.

## Data Availability

Data sharing not applicable to this article as no datasets were generated or analyzed during the current study.

## References

[1] P. K. Mistry , P. Deegan , A. Vellodi , J. A. Cole , M. Yeh , and N. J. Weinreb , “Timing of Initiation of Enzyme Replacement Therapy After Diagnosis of Type 1 Gaucher Disease: Effect on Incidence of Avascular Necrosis,” British Journal of Haematology 147, no. 4 (2009): 561–570. 19732054 10.1111/j.1365-2141.2009.07872.x PMC2774157

[2] S. M. Lo , J. Liu , F. Chen , et al., “Pulmonary Vascular Disease in Gaucher Disease: Clinical Spectrum, Determinants of Phenotype and Long‐Term Outcomes of Therapy,” Journal of Inherited Metabolic Disease 34, no. 3 (2011): 643–650. 21445609 10.1007/s10545-011-9313-9 PMC3782382

[3] M. Kyllerman , N. Conradi , J. E. Månsson , A. K. Percy , and L. Svennerholm , “Rapidly Progressive Type III Gaucher Disease: Deterioration Following Partial Splenectomy,” Acta Paediatrica Scandinavica 79, no. 4 (1990): 448–453. 2349880 10.1111/j.1651-2227.1990.tb11492.x

[4] E. Hertz , Y. Chen , and E. Sidransky , “Gaucher Disease Provides a Unique Window Into Parkinson Disease Pathogenesis,” Nature Reviews Neurology 20, no. 9 (2024): 526–540. 39107435 10.1038/s41582-024-00999-z

[5] G. Bultron , K. Kacena , D. Pearson , et al., “The Risk of Parkinson's Disease in Type 1 Gaucher Disease,” Journal of Inherited Metabolic Disease 33, no. 2 (2010): 167–173. 20177787 10.1007/s10545-010-9055-0 PMC2887303

[6] G. A. Grabowski , A. H. M. Antommaria , E. H. Kolodny , and P. K. Mistry , “Gaucher Disease: Basic and Translational Science Needs for More Complete Therapy and Management,” Molecular Genetics and Metabolism 132, no. 2 (2021): 59–75. 33419694 10.1016/j.ymgme.2020.12.291 PMC8809485

[7] V. Koprivica , D. L. Stone , J. K. Park , et al., “Analysis and Classification of 304 Mutant Alleles in Patients With Type 1 and Type 3 Gaucher Disease,” American Journal of Human Genetics 66, no. 6 (2000): 1777–1786. 10796875 10.1086/302925 PMC1378059

[8] G. A. Diaz , B. D. Gelb , N. Risch , et al., “Gaucher Disease: The Origins of the Ashkenazi Jewish N370S and 84GG Acid Beta‐Glucosidase Mutations,” American Journal of Human Genetics 66, no. 6 (2000): 1821–1832. 10777718 10.1086/302946 PMC1378046

[9] S. Waldman , D. Backenroth , É. Harney , et al., “Genome‐Wide Data From Medieval German Jews Show That the Ashkenazi Founder Event Pre‐Dated the 14(Th) Century,” Cell 185, no. 25 (2022): 4703–4716.e16. 36455558 10.1016/j.cell.2022.11.002 PMC9793425

[10] D. Elstein , N. Belmatoug , B. Bembi , et al., “Twelve Years of the Gaucher Outcomes Survey (GOS): Insights, Achievements, and Lessons Learned From a Global Patient Registry,” Journal of Clinical Medicine 13, no. 12 (2024): 3588. 38930117 10.3390/jcm13123588 PMC11204885

[11] C. S. Boddupalli , S. Nair , G. Belinsky , et al., “Neuroinflammation in Neuronopathic Gaucher Disease: Role of Microglia and NK Cells, Biomarkers, and Response to Substrate Reduction Therapy,” eLife 11 (2022): e79830. 35972072 10.7554/eLife.79830 PMC9381039

[12] S. Nair , C. S. Boddupalli , R. Verma , et al., “Type II NKT‐TFH Cells Against Gaucher Lipids Regulate B‐Cell Immunity and Inflammation,” Blood 125, no. 8 (2015): 1256–1271. 25499455 10.1182/blood-2014-09-600270 PMC4335081

[13] G. Belinsky , J. Ruan , N. Fattahi , et al., “Modeling Bone Marrow Microenvironment and Hematopoietic Dysregulation in Gaucher Disease Through VavCre Mediated Gba Deletion,” Human Molecular Genetics 34, no. 11 (2025): 952–966. 40197748 10.1093/hmg/ddaf045 PMC12085781

[14] M. K. Pandey , T. A. Burrow , R. Rani , et al., “Complement Drives Glucosylceramide Accumulation and Tissue Inflammation in Gaucher Disease,” Nature 543, no. 7643 (2017): 108–112. 28225753 10.1038/nature21368

[15] P. K. Mistry , J. Liu , M. Yang , et al., “Glucocerebrosidase Gene‐Deficient Mouse Recapitulates Gaucher Disease Displaying Cellular and Molecular Dysregulation Beyond the Macrophage,” Proceedings of the National Academy of Sciences of the United States of America 107, no. 45 (2010): 19473–19478. 20962279 10.1073/pnas.1003308107 PMC2984187

[16] M. K. Pandey and G. A. Grabowski , “Immunological Cells and Functions in Gaucher Disease,” Critical Reviews in Oncogenesis 18, no. 3 (2013): 197–220. 23510064 10.1615/critrevoncog.2013004503 PMC3661296

[17] R. Schiffmann , T. M. Cox , J. F. Dedieu , et al., “Venglustat Combined With Imiglucerase for Neurological Disease in Adults With Gaucher Disease Type 3: The LEAP Trial,” Brain 146, no. 2 (2023): 461–474. 36256599 10.1093/brain/awac379 PMC9924909

[18] A. Tylki‐Szymańska , A. Vellodi , A. El‐Beshlawy , J. A. Cole , and E. Kolodny , “Neuronopathic Gaucher Disease: Demographic and Clinical Features of 131 Patients Enrolled in the International Collaborative Gaucher Group Neurological Outcomes Subregistry,” Journal of Inherited Metabolic Disease 33, no. 4 (2010): 339–346. 20084461 10.1007/s10545-009-9009-6

[19] A. El‐Beshlawy , A. Tylki‐Szymanska , A. Vellodi , et al., “Long‐Term Hematological, Visceral, and Growth Outcomes in Children With Gaucher Disease Type 3 Treated With Imiglucerase in the International Collaborative Gaucher Group Gaucher Registry,” Molecular Genetics and Metabolism 120, no. 1–2 (2017): 47–56. 28040394 10.1016/j.ymgme.2016.12.001

[20] T. H. Taddei , K. A. Kacena , M. Yang , et al., “The Underrecognized Progressive Nature of N370S Gaucher Disease and Assessment of Cancer Risk in 403 Patients,” American Journal of Hematology 84, no. 4 (2009): 208–214. 19260119 10.1002/ajh.21362 PMC3008404

[21] J. Sheth , A. Nair , and B. Jee , “Lysosomal Storage Disorders: From Biology to the Clinic With Reference to India,” Lancet Regional Health – Southeast Asia 9 (2023): 100108. 37383036 10.1016/j.lansea.2022.100108 PMC10305895

[22] P. K. Mistry , P. S. Kishnani , M. Balwani , et al., “The Two Substrate Reduction Therapies for Type 1 Gaucher Disease Are Not Equivalent. Comment on Hughes Et al. Switching Between Enzyme Replacement Therapies and Substrate Reduction Therapies in Patients With Gaucher Disease: Data From the Gaucher Outcome Survey (GOS),” Journal of Clinical Medicine 12, no. 9 (2023): 5158. 37176709 10.3390/jcm12093269 PMC10179580

[23] M. Basiri , M. E. Ghaffari , J. Ruan , et al., “Osteonecrosis in Gaucher Disease in the Era of Multiple Therapies: Biomarker Set for Risk Stratification From a Tertiary Referral Center,” eLife 12 (2023): 12. 10.7554/eLife.87537 PMC10317498 37249220

[24] P. K. Mistry , P. Kishnani , C. Wanner , et al., “Rare Lysosomal Disease Registries: Lessons Learned Over Three Decades of Real‐World Evidence,” Orphanet Journal of Rare Diseases 17, no. 1 (2022): 362. 36244992 10.1186/s13023-022-02517-0 PMC9573793

[25] R. Schiffmann , J. Sevigny , A. Rolfs , et al., “The Definition of Neuronopathic Gaucher Disease,” Journal of Inherited Metabolic Disease 43, no. 5 (2020): 1056–1059. 32242941 10.1002/jimd.12235 PMC7540563

[26] A. Donald , S. Brothwell , B. M. Cabello , et al., “250 Cases of “Type 2 Gaucher Disease”: A Novel System of Clinical Categorisation and Evidence of Genotype: Phenotype Correlation,” Molecular Genetics and Metabolism 145, no. 2 (2025): 109124. 40339406 10.1016/j.ymgme.2025.109124

[27] M. Balwani , L. Fuerstman , R. Kornreich , L. Edelmann , and R. J. Desnick , “Type 1 Gaucher Disease: Significant Disease Manifestations in “Asymptomatic” Homozygotes,” Archives of Internal Medicine 170, no. 16 (2010): 1463–1469. 20837833 10.1001/archinternmed.2010.302 PMC3098047

[28] U. Ramaswami , E. Mengel , A. Berrah , et al., “Throwing a Spotlight on Under‐Recognized Manifestations of Gaucher Disease: Pulmonary Involvement, Lymphadenopathy and Gaucheroma,” Molecular Genetics and Metabolism 133, no. 4 (2021): 335–344. 34229967 10.1016/j.ymgme.2021.06.009

[29] V. Murugesan , W. L. Chuang , J. Liu , et al., “Glucosylsphingosine Is a Key Biomarker of Gaucher Disease,” American Journal of Hematology 91, no. 11 (2016): 1082–1089. 27441734 10.1002/ajh.24491 PMC5234703

[30] P. K. Mistry , N. J. Weinreb , P. Kaplan , J. A. Cole , A. R. Gwosdow , and T. Hangartner , “Osteopenia in Gaucher Disease Develops Early in Life: Response to Imiglucerase Enzyme Therapy in Children, Adolescents and Adults,” Blood Cells, Molecules & Diseases 46, no. 1 (2011): 66–72. 10.1016/j.bcmd.2010.10.011 PMC3019260 21112800

[31] B. E. Rosenbloom , M. D. Cappellini , N. J. Weinreb , et al., “Cancer Risk and Gammopathies in 2123 Adults With Gaucher Disease Type 1 in the International Gaucher Group Gaucher Registry,” American Journal of Hematology 97, no. 10 (2022): 1337–1347. 36054609 10.1002/ajh.26675 PMC9541044

[32] Y. V. Taguchi , J. Liu , J. Ruan , et al., “Glucosylsphingosine Promotes α‐Synuclein Pathology in Mutant GBA‐Associated Parkinson's Disease,” Journal of Neuroscience 37, no. 40 (2017): 9617–9631. 28847804 10.1523/JNEUROSCI.1525-17.2017 PMC5628407

[33] C. Blauwendraat , N. Tayebi , E. G. Woo , et al., “Polygenic Parkinson's Disease Genetic Risk Score as Risk Modifier of Parkinsonism in Gaucher Disease,” Movement Disorders 38, no. 5 (2023): 899–903. 36869417 10.1002/mds.29342 PMC10271962

[34] E. G. Woo , N. Tayebi , and E. Sidransky , “Next‐Generation Sequencing Analysis of GBA1: The Challenge of Detecting Complex Recombinant Alleles,” Frontiers in Genetics 12 (2021): 684067. 34234814 10.3389/fgene.2021.684067 PMC8255797

[35] S. R. L. Vieira , R. Mezabrovschi , M. Toffoli , et al., “Consensus Guidance for Genetic Counseling in GBA1 Variants: A Focus on Parkinson's Disease,” Movement Disorders 39, no. 12 (2024): 2144–2154. 39258449 10.1002/mds.30006 PMC11657020

[36] H. Reichmann , I. Csoti , J. Koschel , et al., “Life Style and Parkinson's Disease,” Journal of Neural Transmission 129, no. 9 (2022): 1235–1245. 35606622 10.1007/s00702-022-02509-1 PMC9463300

[37] M. M. McGovern , M. P. Wasserstein , R. Giugliani , et al., “A Prospective, Cross‐Sectional Survey Study of the Natural History of Niemann‐Pick Disease Type B,” Pediatrics 122, no. 2 (2008): e341–e349. 18625664 10.1542/peds.2007-3016 PMC2692309

[38] M. Nickel and A. Schulz , “Natural History Studies in NCL and Their Expanding Role in Drug Development: Experiences From CLN2 Disease and Relevance for Clinical Trials,” Frontiers in Neurology 13 (2022): 785841. 35211079 10.3389/fneur.2022.785841 PMC8861081

[39] D. Elstein , M. W. Klutstein , A. Lahad , A. Abrahamov , I. Hadas‐Halpern , and A. Zimran , “Echocardiographic Assessment of Pulmonary Hypertension in Gaucher's Disease,” Lancet 351, no. 9115 (1998): 1544–1546. 10326537 10.1016/S0140-6736(98)10194-0

[40] P. K. Mistry , N. J. Weinreb , R. O. Brady , and G. A. Grabowski , “Gaucher Disease: Resetting the Clinical and Scientific Agenda,” American Journal of Hematology 84, no. 4 (2009): 205–207. 19296473 10.1002/ajh.21384 PMC2999876

[41] P. K. Mistry , J. L. Batista , H. C. Andersson , et al., “Transformation in Pretreatment Manifestations of Gaucher Disease Type 1 During Two Decades of Alglucerase/Imiglucerase Enzyme Replacement Therapy in the International Collaborative Gaucher Group (ICGG) Gaucher Registry,” American Journal of Hematology 92, no. 9 (2017): 929–939. 28569047 10.1002/ajh.24801 PMC5600096

[42] G. A. Grabowski , K. Kacena , J. A. Cole , et al., “Dose‐Response Relationships for Enzyme Replacement Therapy With Imiglucerase/Alglucerase in Patients With Gaucher Disease Type 1,” Genetics in Medicine 11, no. 2 (2009): 92–100. 19265748 10.1097/GIM.0b013e31818e2c19 PMC3793250

[43] D. Amato , T. Stachiw , J. T. Clarke , and G. E. Rivard , “Gaucher Disease: Variability in Phenotype Among Siblings,” Journal of Inherited Metabolic Disease 27, no. 5 (2004): 659–669. 15669682 10.1023/b:boli.0000042983.60840.f3

[44] G. A. Diaz , R. Giugliani , N. Guffon , et al., “Long‐Term Safety and Clinical Outcomes of Olipudase Alfa Enzyme Replacement Therapy in Pediatric Patients With Acid Sphingomyelinase Deficiency: Two‐Year Results,” Orphanet Journal of Rare Diseases 17, no. 1 (2022): 437. 36517856 10.1186/s13023-022-02587-0 PMC9749157

[45] S. Jones , M. Coker , A. G. López , et al., “Open‐Label Phase 1/2 Study of Vestronidase Alfa for Mucopolysaccharidosis VII,” Molecular Genetics and Metabolism Reports 28 (2021): 100774. 34136357 10.1016/j.ymgmr.2021.100774 PMC8178115

[46] S. A. Jones , S. Rojas‐Caro , A. G. Quinn , et al., “Survival in Infants Treated With Sebelipase Alfa for Lysosomal Acid Lipase Deficiency: An Open‐Label, Multicenter, Dose‐Escalation Study,” Orphanet Journal of Rare Diseases 12, no. 1 (2017): 25. 28179030 10.1186/s13023-017-0587-3 PMC5299659

[47] M. Nicolino , B. Byrne , J. E. Wraith , et al., “Clinical Outcomes After Long‐Term Treatment With Alglucosidase Alfa in Infants and Children With Advanced Pompe Disease,” Genetics in Medicine 11, no. 3 (2009): 210–219. 19287243 10.1097/GIM.0b013e31819d0996

[48] Y. B. Sohn , A. Yang , M.‐S. Kim , et al., “Efficacy and Safety of Idursulfase Beta in the Treatment of Mucopolysaccharidosis II: A Phase‐3, 2‐Part Study Compared With a Historical Placebo Cohort,” Genetics in Medicine 27, no. 8 (2025): 101460. 40411345 10.1016/j.gim.2025.101460

[49] M. K. Pandey , G. A. Grabowski , and J. Köhl , “An Unexpected Player in Gaucher Disease: The Multiple Roles of Complement in Disease Development,” Seminars in Immunology 37 (2018): 30–42. 29478824 10.1016/j.smim.2018.02.006

